# Three-dimensional computed tomographic angular measurements of the canine tibia using a bone-centered coordinate system

**DOI:** 10.3389/fvets.2023.1154144

**Published:** 2023-05-30

**Authors:** Andreas Brühschwein, Bronson Schmitz, Martin Zöllner, Sven Reese, Andrea Meyer-Lindenberg

**Affiliations:** ^1^Clinic of Small Animal Surgery and Reproduction, Centre of Veterinary Clinical Medicine, Faculty of Veterinary Medicine, LMU Munich, Munich, Germany; ^2^Institute of Veterinary Anatomy, Histology and Embryology, Department of Veterinary Sciences, Faculty of Veterinary Medicine, LMU Munich, Munich, Germany

**Keywords:** dog, tibia, computed tomography, 3D coordinate system, angle measurement, angular deformity, torsion, varus

## Abstract

**Introduction:**

Canine tibial alignment is determined by two-dimensional angular measurements, and tibial torsion is challenging. Aim of the study was the development and evaluation of a CT technique to measure canine tibial varus and torsion angles independent from positioning and truly three-dimensional.

**Materials and methods:**

A bone-centered 3D cartesian coordinate system was introduced into the CT-scans of canine tibiae and aligned with the anatomical planes of the bone based on osseous reference points. Tibial torsion, and varus (or valgus) angles were calculated based on geometric definition of projection planes with VoXim® medical imaging software using 3D coordinates of the reference points. To test accuracy of the tibial torsion angle measurements, CT scans of a tibial torsion model were performed in 12 different hinge rotation setups ranging from the normal anatomical situation up to +/ 90° and compared to goniometer measurements. Independency of tibial positioning on the CT scanner table was evaluated in 20 normal canine tibiae that were scanned in a position parallel to the z-axis and two additional off-angle double oblique positions having 15° and 45° deviation in direction of the x- and y-axes. Angular measurements in oblique positions were compared with the normal parallel position by subtraction. Precision was tested using clinical CT scans of 34 canine patients with a clinical diagnosis of patellar luxation.

**Results:**

Accuracy testing in the tibial torsional deformity model revealed a difference of 0.2° demonstrated by Passing-Bablok analysis and Bland–Altman-Plots. Testing for independency from tibial positioning resulted in mean differences less than 1.3°. Precision testing in clinical patients resulted in coefficients of variation for repeated measurements of 2.35% (intraobserver agreement) and 0.60% (interobserver agreement) for the tibial torsion angle, and 2.70% (intraobserver agreement) and 0.97% (interobserver agreement) for the tibial varus (or valgus) angle.

**Discussion:**

The technique is lacking determination of bone deformities in the sagittal plane, and demonstration of accuracy in severe complex bone deformities in multiple planes.

In conclusion, we developed a method to measure canine tibial torsional and varus or valgus deformities, that calculates in 3D space, and we demonstrated its accuracy in a torsional deformity model, and its precision in CT data of clinical patients.

## 1. Introduction

Radiography of the canine tibia is commonly performed to diagnose and quantify osseous deformity using angular measurements (1–8). Radiography is the reduction of a three-dimensional (3D) object into a two-dimensional image by superimposition of all structures along the path of the x-ray beam (9). Due to the projection of the object into a single plane, geometric error occurs as a result of magnification and distorsion (9). Distorsion is unequal magnification that leads to a radiographic image that does not truly represent the real shape and size of the examined object (9). Positioning and intrinsic morphologic variation and of the object, as well as x-ray beam centering and angulation contributes to the variation of the radiographic shape and image of a canine tibia. In geometry, two points define a line and an angle is formed by two, not parallel, intersecting lines within the same plane, that is called coplanarity (10). In radiography, angular measurements of bones are based on osseous reference points that are projected into a two-dimensional summation image, but in three-dimensional anatomic reality theses points are not coplanar (9, 10). Radiographic projection creates coplanarity in the radiographic image, that commonly is not present on the object, due to the three-dimensional nature of anatomy. Reference points that are not coplanar define axes that are skew lines in the patient (10, 11). Skew lines are not parallel and not coplanar (11). Instead, skew lines are in separate planes, share no intersection and do not form an angle in three-dimensional geometry (11). Angles measured on a radiograph are commonly formed by skew lines in the patient that are projected in to a common shared image plane. To overcome radiographic limitations of geometric distortion and to minimize measurement error that is caused by the variation of projection of skew lines into the image plane, standardization of positioning as well as beam centering and alignment during radiography plays an important role (7). Therefore, two-dimensional radiographic angular measurement techniques heavily rely on standardized positioning and projection, that can be easily achieved in normal bones or normal cadavers, but can become challenging in animals that are suffering from angular limb deformities or orthopedic disorders with restricted articular range of motion.

Limb malalignments are described in relation to the three orthogonal anatomical planes: sagittal, transverse and dorsal [frontal] (12). The median plane divides the body symmetrically along its longitudinal axis into left and right halves (13, 14). The sagittal plane is parallel to the median and paramedian planes (13, 14). The transverse plane is perpendicular to the longitudinal axis of the body or its parts, such as the limbs (13, 14). The dorsal plane is parallel to the dorsal surface of the body or its parts, and perpendicular to the sagittal and transverse planes (13, 14). In the limb, the term “*frontal*” has also been used in the past as a synonym for” *dorsal*,” which is not in accordance with the current Nomina Anatomica Veterinaria (NAV) (13). Tibial alignment in the dorsal plane is determined on craniocaudal radiographs and abnormal bending in the lateromedial or mediolateral direction is called varus or valgus deformity, respectively (1, 3, 5–8, 12, 15, 16). Twisting of the tibia around its longitudinal diaphyseal axis in transverse plane is called tibial torsion (2, 12, 17–20). Flexion and extension of the canine stifle and tarsal joint mainly occur in the sagittal plane of the limb and abnormal tibial torsion might cause deviation between the flexion-extension plane of the stifle and the talocrural joint. To characterize canine hind limb deformities and patellar luxation more specifically, determination of tibial torsion was investigated using radiography and computed tomography (2, 17, 18). Radiographic determination of tibial torsion on caudocranial radiographs can be estimated by comparing the appearance and shape of proximal and distal aspects of the bone (12) or uses the calcaneus as tarsal and distal femoral sesamoid bones as femoral landmarks (2, 19). Use of articulating, but extra-tibial reference points introduce potential sources of errors, that include intra-articular femorotibial and tibiotalar joint rotation as well as luxation, displacement, deformity or fracture of patellar and fabellar sesamoid bones (21–25).

Computed tomography (CT) is able to visualize tibial landmarks directly to determine the angular relationship between the proximal and distal articular orientation around the longitudinal tibial axis to calculate tibial torsion in the human (26–30) and canine patient (2, 17, 18, 20, 31, 32). Computed tomographic determination of tibial torsion requires standardized positioning of the tibia parallel to the z-axis of the scanner and was established in disarticulated cadaver hindlimbs (2, 17). In a study focusing on determination of tibial torsion from CT scans during a clinical setting the reference points were not within the same CT image and reference axes were determined between several slices and angles were measured indirectly to the horizontal image border (18). In human medicine computed tomographic determination of tibial torsion is erroneous, if the tibia is not totally straight positioned in the scanner causing oblique cross-sectional images and therefore it is recommended to realign the scan using multiplanar reconstruction (MPR) prior to the measurement, that is based on summation of two two-dimensional images (30). In humans, the normal vertical standing and horizontal lying positing in the CT scanner is similar. CT of the awake standing small dog is described (33), but more commonly the quadruped canine patient is scanned in dorsal recumbency with extended coxofemoral joints and caudally positioned hindlimbs (18, 20, 34–37). Different types of positioning are described. Various positions of the hind limbs are described with the hocks and stifles, each flexed at 90° to full joint extension (18, 20, 34–37). Even if straight and extended positing is aimed, most canine coxofemoral joints and stifles cannot be fully extended to 180° and therefore tibia and femur, both at the same time cannot be positioned parallel to the z-axis of the scanner table. Additionally, bone deformity due to malformation or fracture malunion, limited articular range of motion due to osteoarthritis or muscular contractions might impede or prevent standardized positioning. In a clinical setting, precise determination of three-dimensional limb alignment is especially interesting in patients with complex limb deformities, where angular measurements of tibia and femur in both hindlimbs might be of interest, for example in dogs with patellar luxation, where in addition to soft tissue abnormalities concomitant femoral and tibial deformations might play a role (18, 31, 32, 34–36, 38).

CT acquires 3D data, but if osseous reference points for angular measurements are located in different CT images, postprocessing of the cross-sectional images is required. Angular measurements can be performed within images that are created by summation or superimposition of two or more individual CT images into one single superimposed image (2, 17, 18, 20, 37), using MPR (34–37), maximum intensity projections (MIP) (37) or volume rendering technique (VR) (20, 36–38). MPR, MIP or VR allow free rotation and free choice of perspective, that is considered three-dimensionality. However, based on the final selected view, a planar image is created that lacks a third dimension. Projection error remains, because points and lines that define the angle are coplanar in the postprocessed and reconstructed CT image but remain skew lines in the patient. Angular measurements in complex angular and torsional deformities are still limited despite the use of VR CT (39). To standardize viewing and measurement perspective in CT scans of normal canine cadaver tibiae a combination of MPR, fiducial markers in the mechanical axis, proximodistal VR-views and semitransparent bone filter were introduced (20). Despite use of the three-dimensionality of computed tomography, the majority of reported techniques are restricted to perfect tibial positioning, determine angular measurements within a single two-dimensional image only, have limitations in the three-dimensional geometric definition of the projection of skew lines into the measurement planes, or are described or evaluated in isolated or normal physiological bones only.

Therefore, goals of our study were to develop a method to measure canine tibial torsional and varus or valgus deformities, that is independent from positioning, that includes a precise 3D geometrical definition of skew line projection into angular measurement planes, to test its accuracy in a torsional deformity model, to test its independency of tibial positioning on the scanner table, and to test its precision in CT data of clinical canine patients with orthopedic hind limb disorders and presumed osseous deformation.

## 2. Materials and methods

### 2.1. Development of the technique

#### 2.1.1. Prototyping

For prototyping and initial testing of the program and software templates we queried the picture archiving and communication system (dicomPACS, Oehm & Rehbein, Rostock, Germany) of the hospital and retrieved a CT study with presumably normal canine tibiae of a large-bred mixed breed dog from a patient that was scanned for clinical reasons unrelated to the tibiae in a position that was similar to a ventrodorsal pelvic radiograph for canine hip dysplasia screening, having extended coxofemoral, stifle and tarsal joints with the hind limbs’ longitudinal axes parallel to the lumbar spine and used a copy of the images. We used images reconstructed with of 0.6 mm slice thickness without interslice gap reconstructed with a high-frequency reconstruction algorithm (bone filter, kernel 70).

#### 2.1.2. Software

For the software calculations and measurements of this study, we used the software, VoXim® (version 6.5.1.1 (T2160910) Copyright©) from the medical imaging company IVS Technology GmbH [LLC], Chemnitz, Germany, that met our inclusion criteria. The standard medical imaging software VoXim® was conformant with the DICOM (Digital Imaging and Communications in Medicine) technical standard, had medical device approval, and was designed for 3D measurements and surgical planning (40–43). Fixed orthogonal MPR in a bone window, VR, segmentation of bones, variable three-dimensional coordinate systems, vector-based measurement tools and the adjustment of templates for predefined calculations were the main features. For the purpose of this study, the software plugins were specially adapted to our requirements by one of the software engineers of the company. To measure angles in the canine tibia in a way that is truly three-dimensional, a bone-centered 3D coordinate system was introduced based on anatomical reference points to define anatomical axes and lines precisely within the 3D space of a stack of continuous gap-free CT slices, as described for the canine femur (44). Anatomical reference points defining anatomical axes that were skew lines were projected into geometrically predefined projection planes. Angular measurements were enabled by 3D vector calculations of the software based on the 3D coordinates of the individual reference points. Tibial reference points and axes described by radiography and CT in a two-dimensional way were extended into a detailed three-dimensional anatomical description using VR and orthogonal three plane MPR. Reference points were adjusted and new reference points and axes were introduced, where required. Mathematical definitions of the projection planes were defined by reference axes and were inspired by anatomic cross-section planes, radiographic image planes and x-ray beam projection techniques as well as computed tomographic VR-views.

### 2.2. Description of reference points, axes, planes, coordinate system, and calculation of the angles

#### 2.2.1. Tibial torsion angle

**Figure 1:**
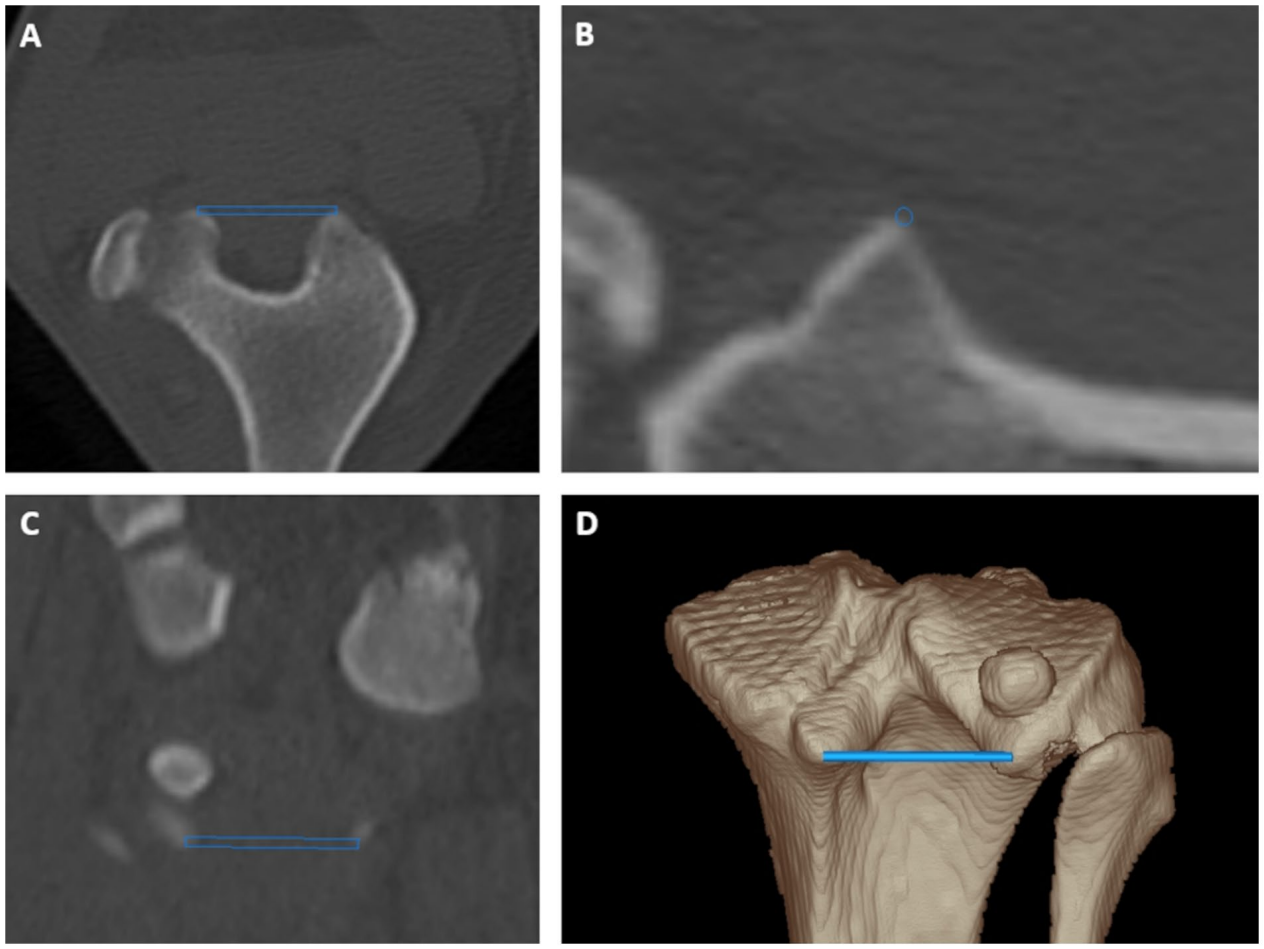
Tibial torsion angle: proximal caudal tibial retrocondylar axis was a mediolaterally running tangent located caudally to the tibial head and tibial plateau connecting both most prominent caudal protrusions of the medial and lateral tibial condyle in transverse **(A)**, sagittal **(B)** and dorsal **(C)** plane multiplanar reformation and a 3D volume rendering view **(D)** from a caudoproximal perspective.

**Figure 2:**
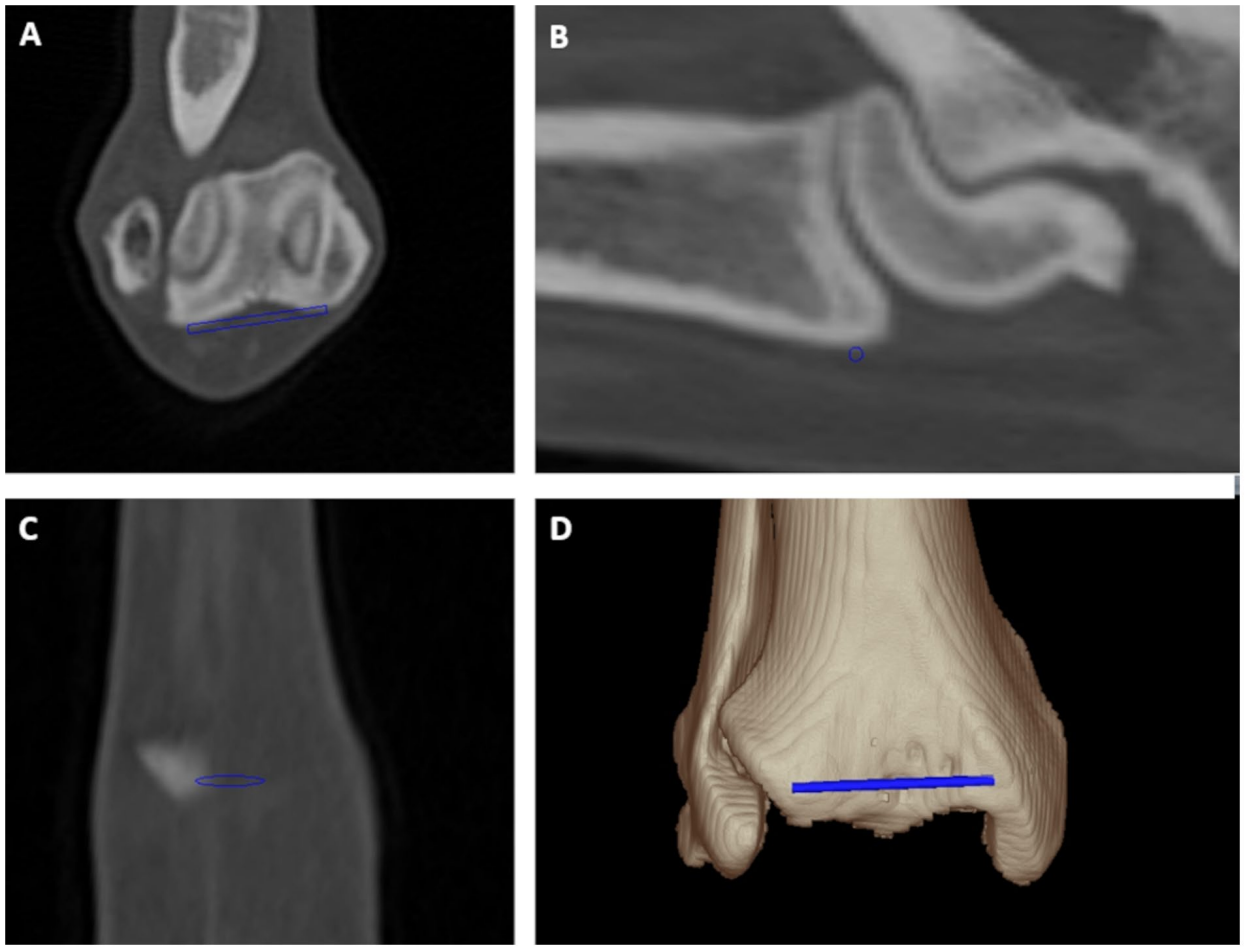
Tibial torsion angle: distal cranial tibial antecondylar axis was a mediolaterally running tangent located cranially to the tibial cochlea connecting both most prominent cranial medial and lateral bony protrusions at bone surface proximally and parallel to the tibiotalar joint in transverse **(A)**, sagittal **(B)** and dorsal **(C)** plane multiplanar reformation and a 3D volume rendering view **(D)** from a cranial perspective.

**Figure 3:**
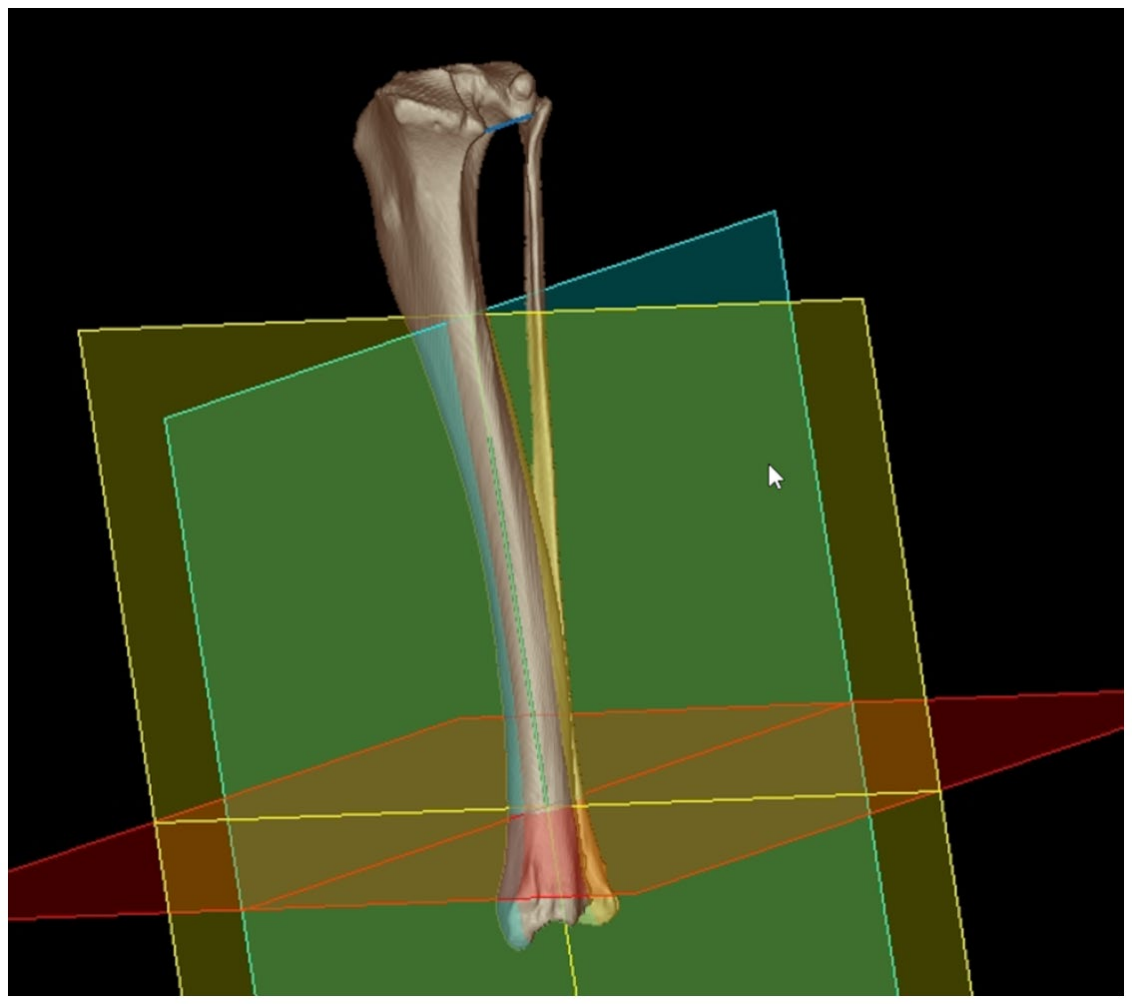
Bone-centered 3D coordinate system: the distal tibial center was the point of origin for an orthogonal 3D Cartesian coordinate system with the total tibial longitudinal axis (green) as the first axis that defines the transverse plane (red) by orthogonality. The retrocondylar axis (blue line proximally) was translated to the total tibial longitudinal axis and defined the orientation of the dorsal plane (blue/green) as the second axis. The third orthogonal axis corresponded to the orientation of the sagittal plane (yellow). Introduction of the bone-centered 3D coordinate system having orthogonal x-, y-, and z-axes enabled mathematical definition of the projection planes and angular calculations based on vector geometry using 3D coordinates.

For the tibial torsion angle (TTA), caudal to the tibial head and caudodistal to the tibial plateau, a mediolateral transverse tangent was aligned by setting two points abaxially at the caudal surface of the proximal tibial cortex along the two most prominent protrusions of the medial (Supplementary Figure S1) and lateral tibial condyle (Supplementary Figure S2) that defined the proximal caudal tibial retrocondylar axis (Figure 1). Cranial to the tibial cochlea and proximal to the talocrural joint space, a transverse tangent was aligned by setting two points abaxially at the cranial surface of the distal tibial cortex at the furthermost prominent medial (Supplementary Figure S3) and lateral bony protrusion (Supplementary Figure S4) that defined the distal cranial tibial antecochlear axis (Figure 2). To calculate the angles based on vector geometry a bone-centered coordinate system was introduced. The proximal caudal tibial axis and the distal cranial tibial axis were skew lines. For the calculation of the tibial torsion angle, they required projection by parallel translation into the same transverse plane, that was defined by perpendicularity to a total tibial longitudinal axis defined by two reference points along the tibial diaphysis (Supplementary Figure S5). For this purpose, the proximal tibial shaft center (PTC) was placed using an encircled crossline tool in the midpoint of the proximal tibial diaphysis at the level of the distal end of the tibial crest and the tibial nutrient foramen where the proximal triangular appearance of the tibial transverse cross section became round, scrolling proximo-distally. In the same way the distal tibial shaft center (DTC) was placed in the midpoint of the distal tibial diaphysis, at the level where the distal semioval to triangular appearance of the tibial transverse cross section turned into a round cross-sectional shape, scrolling disto-proximally to the diaphysis (Supplementary Figure S5). The point of origin of the tibial bone-centered coordinate system (Figure 3) was the distal tibial shaft center. The distal and proximal tibial shaft centers defined the first axis (total tibial longitudinal axis) of the bone-centered tibial coordinate system. The transverse projection plane was perpendicular to the total tibial longitudinal axis. The second axis corresponded to the orientation of the dorsal plane of the bone and was defined by parallel translation of the proximal caudal tibial retrocondylar axis. The sagittal plane was perpendicular to the dorsal and transverse plane (Figure 3). All three planes intersected orthogonally at the level of the distal tibial shaft center, that was the point of origin for a fully three-dimensional Cartesian coordinate system having x-, y- and z-axes, defining each point in the three-dimensional space by means of three coordinates and allowing automatic angular calculations based on vector geometry by the medical imaging software VoXim®.

#### 2.2.2. Tibial varus or valgus angle

**Figure 4:**
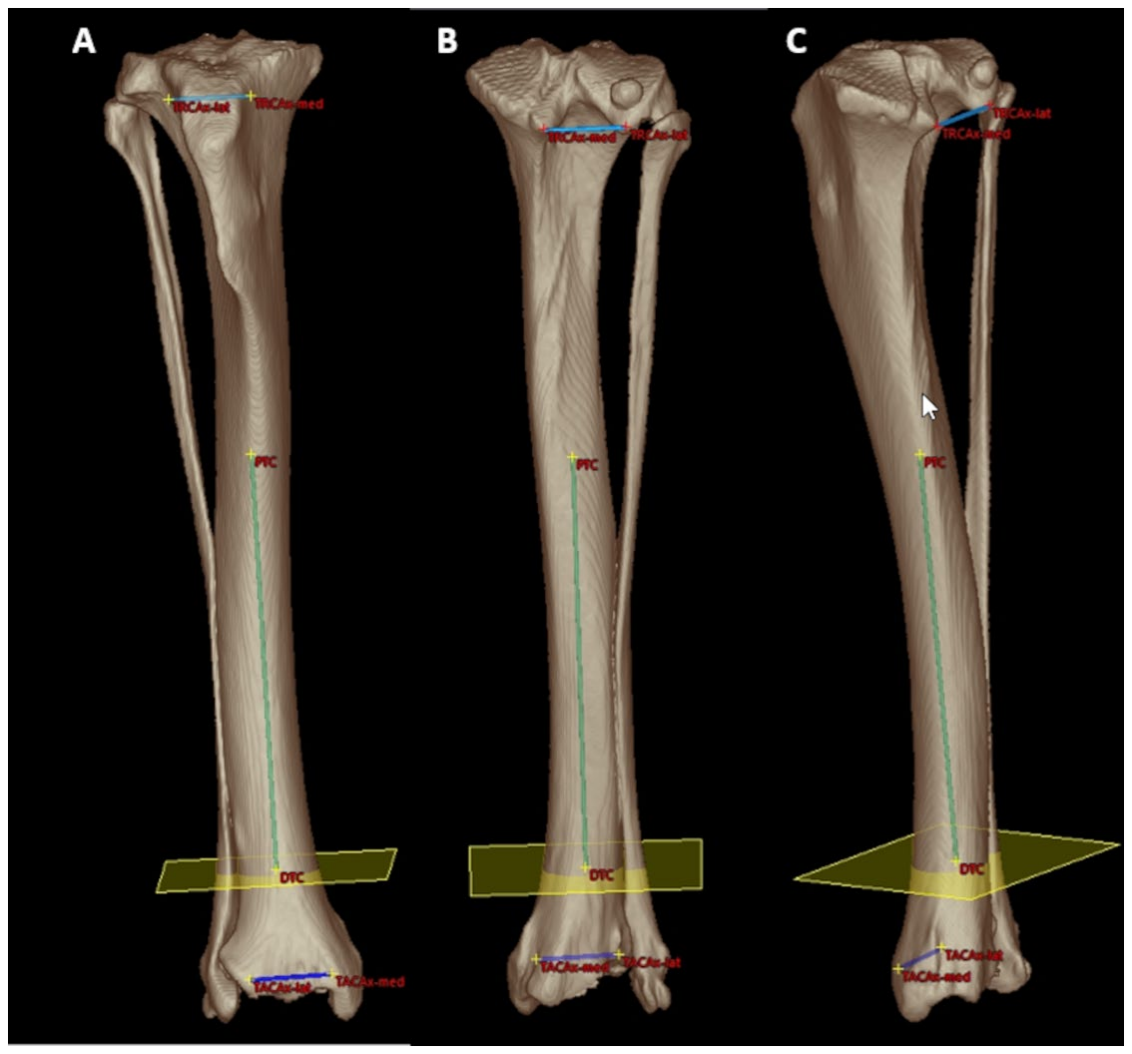
Mathematical definition of the projection plane for the tibial torsion angle: cranial **(A)**, caudal **(B)** and caudomedial **(C)** 3D volume rendering views of a right canine tibia with overlays of the proximal caudal tibial retrocondylar axis (proximal mediolateral light blue line between TRCAx-med and TRCAx-lat) and the distal cranial tibial antecochlear axis (distal mediolateral dark blue line between TACAx-med and TACAx-lat). Both axes were skew lines and required projection into the transverse (yellow) plane mathematically defined by orthogonality to the total tibial longitudinal axis (green diaphyseal line between the distal (DTC) and proximal (PTC) tibial center) to enable angular measurements. Reference points and axes located within or behind bone were lucently projected on the image front overlaying the semitransparent bone surface seen on a brightened color coding **(A–C)**.

**Figure 5:**
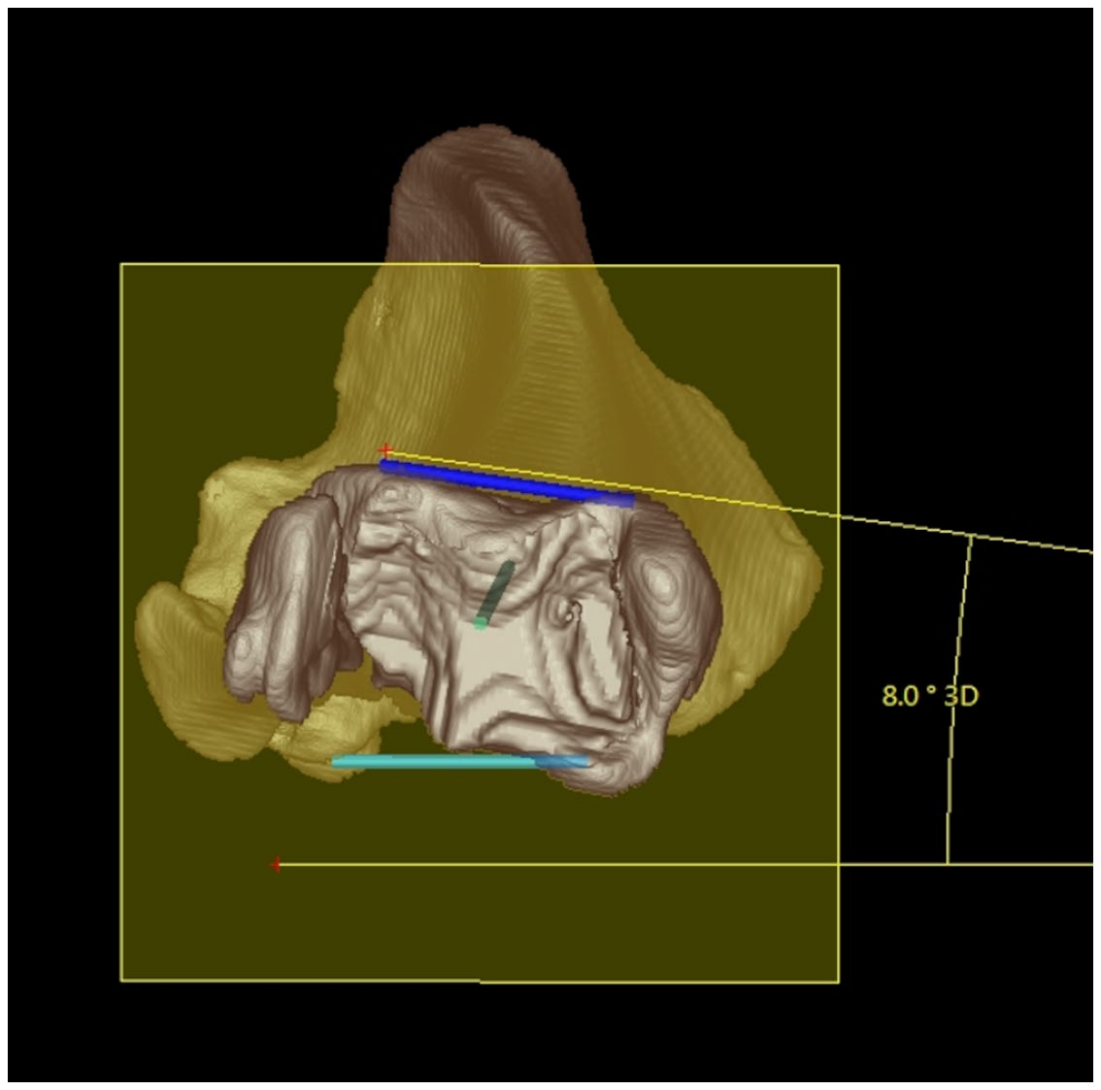
Tibial torsion angle: distal 3D semitransparent volume rendering view of a right canine tibia. The tibial torsion angle (in this example 8°) was determined between the proximal caudal tibial retrocondylar axis (mediolateral light blue line) and the distal cranial tibial antecochlear axis (mediolateral dark blue line) that were both projected in into the transverse (yellow) plane that is determined by orthogonality to the total tibial longitudinal axis (green intraosseous proximodistal diaphyseal line) to enable and define angular measurements between skew lines.

**Figure 6:**
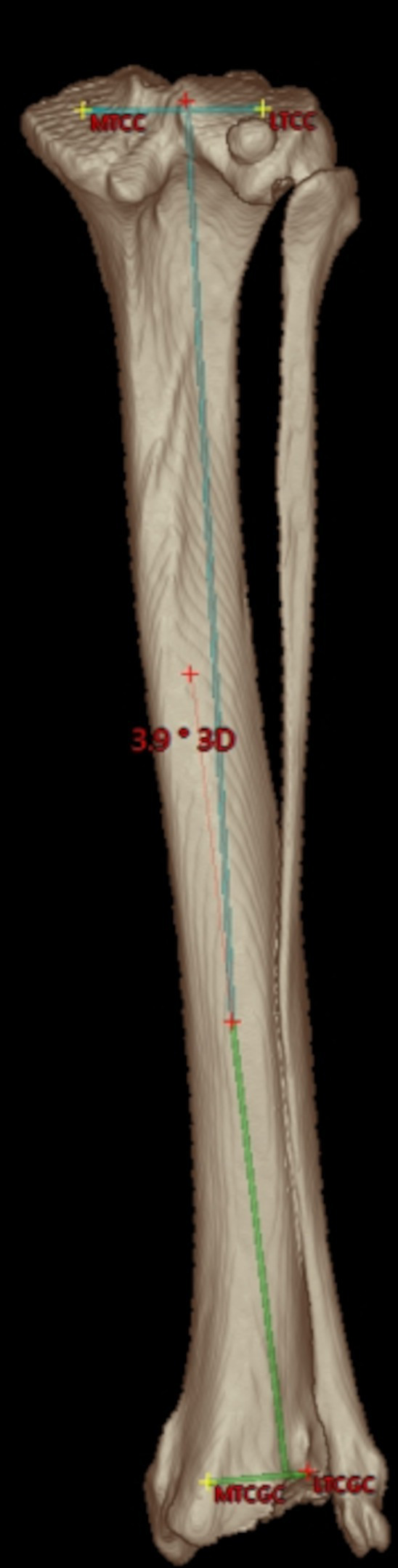
Tibial varus or valgus angle: caudal semitransparent 3D volume rendering view of a right canine tibia with overlays of the proximal and distal tibial mediolateral joint surface axes that are projected into the dorsal tibial plane as defined by the tibial coordinate system. Proximally, the medial (MTCC) and lateral tibial condyle center (LTCC) define the orientation of the proximal tibial joint surface orientation line. Distally, the medial (MTCGC) and lateral tibial cochlear groove center (LTCGC) define the orientation of the distal tibial joint surface orientation line. Orthogonal plumb lines to the proximal and distal tibial mediolateral joint surface axes projected in the dorsal plane define the proximal (light blue) and distal (light green) tibial longitudinal axes and the direction of the angular opening at their intersection correspond to a varus or valgus conformation. In this example between the proximal and distal longitudinal tibial axes, an acute angle of 3.9° is shown. Lateral deviation of the distal axis indicates a valgus angle of 3.9°.

For the tibial varus (or valgus) angle (TVA), proximal and distal the tibial mediolateral joint surface axes were determined. The proximal tibial mediolateral joint surface axis was defined by two points, which were set in the lateral (LTCC) and medial tibial condyle center (MTCC; Supplementary Figure S6). Images in transverse plane (Supplementary Figure S6A) showed the reference points at the joint surface appearing indistinct due to partial volume effect. In sagittal plane reconstructed images, the tibial condyle centers represented the craniocaudal midpoint of the mildly convex surface of each condyle (Supplementary Figure S6B). In dorsal plane reconstructed images, these points corresponded to the mediolateral midpoint and deepest center of the concave surface of each condyle (Supplementary Figure S6C). In a proximodistal three-dimensional volume rendering view at the proximal tibial surface, the points visually reflected the centers of the proximal articular surfaces of the lateral and medial tibial condyle (Supplementary Figure S6D). The distal tibial mediolateral joint surface axis was defined by the lateral (LTCGC) and medial tibial cochlear groove center (MTCGC) of the distal tibial articular surface (Supplementary Figure S7). In transverse reconstructed images, the furthermost proximal slice of the tibiotalar joint space proximally to the talar ridges defined the subchondral bone of the medial and lateral cochlear groove centers (Supplementary Figure S7A). In sagittal plane reconstructed images, the deepest impression and craniocaudal tibial midpoint reflected the medial and lateral cochlear groove centers (Supplementary Figure S7B). In dorsal plane reconstructed images, these points corresponded to the mediolateral midpoint and deepest center of the concave surface of the medial and lateral cochlear groove (Supplementary Figure S7C). In a distoproximal three-dimensional volume rendering view at the distal tibial surface, the points reflect the centers of the medial and lateral cochlear groove (Supplementary Figure S7D). For the calculation of the tibial torsion angle, the proximal caudal tibial axis and the distal cranial tibial axis, that were skew lines, needed to intersect. For parallel translation of the proximal and distal axes, the software dropped perpendiculars from the proximal and distal tibial torsion reference points into the transverse projection plane (Figure 4). The angle between the proximal caudal tibial axis and the distal cranial tibial axis projected in the transverse projection plane corresponded to the tibial torsion angle (Figure 5). For the calculation of the tibial varus (or valgus) angle, perpendicular projections translated the proximal and distal tibial mediolateral joint surface axes into the dorsal projection plane (Figures 3, 6). Orthogonal plumb lines to the proximal and distal tibial mediolateral joint surface axes defined the proximal and the distal tibial longitudinal axes. The direction of the angular opening at their intersection between both axes defined the tibial varus (or valgus) conformation (Figure 6). Lateral deviation of the distal axis indicated a valgus and medial deviation the varus angle. In the mediolateral axial center of the tibia, the proximal and distal tibial longitudinal axes resembled radiographic proximal and distal mechanical axes.

#### 2.2.3. Angle readout

There was no tibial torsion, if the proximal caudal tibial retrocondylar axis and the distal cranial tibial antecochlear axis were parallel to each other. There was no varus or valgus, if the proximal and distal tibial longitudinal axes were parallel to each other. This neutral state was read out as 180° on the goniometer and on the software for both the torsion and valgus angles. As the sum of two adjacent supplementary angles is 180°, and to define the direction of axis deviation, the angle measurements were normalized to 180° during the readout. In the transverse plane angle measuring disc, tibial torsion angles were less than 180°, if there was internal rotation of the distal cranial tibia antecochlear axis, relative to the proximal caudal tibial retrocondylar axis as the proximal base. Angles greater than 180° denote external rotation of the distal cranial tibial antechochlear axis with respect to the proximal caudal tibial retrocondylar axis. In the dorsal plane angle measuring disc, valgus angles were read out greater than 180°, and varus angles were smaller than 180°. Names and abbreviations of reference points, axes, angles and planes with related Figures are summarized in Table 1.

**Table 1:** Summary of reference points, axes, angles, planes with abbreviations and related figures.

Reference points	
Proximal caudal tibial retrocondylar axis - medial reference point (TRCAx-med)	Supplementary Figure S1
Proximal caudal tibial retrocondylar axis - lateral reference point (TRCAx-lat)	Supplementary Figure S2
Distal cranial tibial antecochlear axis - medial reference point (TACAx-med)	Supplementary Figure S3
Distal cranial tibial antecochlear axis - lateral reference point (TACAx-lat)	Supplementary Figure S4
Distal tibial shaft center (DTC)	Supplementary Figure S5
Proximal tibial shaft center (PTC)	Supplementary Figure S5
Lateral tibial condyle center (LTCC)	Supplementary Figure S6
Medial tibial condyle center (MTCC)	Supplementary Figure S6
Lateral tibial cochlear groove center (LTCGC)	Supplementary Figure S7
Medial tibial cochlear groove center (MTCGC)	Supplementary Figure S7
Axes	
Proximal caudal tibial retrocondylar axis (TRCAx)	Figure 1
Distal cranial tibial antecochlear axis (TACAx)	Figure 2
Proximal tibial mediolateral joint surface axis	Figure 6, Supplementary Figure S6
Distal tibial mediolateral joint surface axis	Figure 6, Supplementary Figure S7
Total tibial longitudinal axis	Figures 3–5, Supplementary Figure S5
Proximal tibial longitudinal axis	Figure 6
Distal tibial longitudinal axis	Figure 6
Angles	
Tibial torsion angle (TTA)	Figure 5
Tibial varus (or valgus) angle (TVA)	Figure 6
Coordinate system and planes	
Tibial coordinate system	Figure 3
Dorsal projection plane	Figures 3, 6
Transverse projection plane	Figures 3–5

### 2.3. Evaluation of the technique

#### 2.3.1. Accuracy in a tibial torsional deformity model

**Figure 7:**
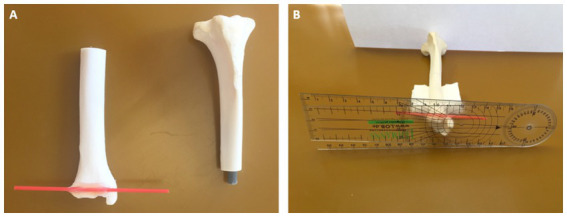
Tibial torsional deformity model to evaluate accuracy and interobserver variability (reproducibility): a physiologic canine tibia was cut in transverse plane at mid-diaphyseal level and a fitting cylinder was introduced into the round medullary cavity that provided a rotational hinge and enabled free rotation of both segments about the tibial diaphyseal longitudinal axis **(A)**. Along the distal cranial tibial antecochlear axis, a thin red plastic pin was precisely glued according to the reference points in transverse axis tangentially at the distal bone surface **(A,B)**. With the caudal prominences of both tibial condyles recumbent on the ground of a flat horizontal surface, corresponding to the proximal caudal tibial retrocondylar axis, and the diaphysis elevated with the longitudinal axis horizontally using supportive material **(B)**, preset tibial torsion angles were measured between the ground corresponding to the proximal caudal tibial retrocondylar axis, and the red plastic pin corresponding to the distal cranial tibial antecochlear axis, using a goniometer (universal manual transparent plastic full circle 360° with 1° readout increment, Rulongmeter style).

Accuracy and interobserver variability (reproducibility) were tested in a torsional deformity model (Figure 7). A physiologic canine tibia of a medium sized dog, received from the teaching material collection of the veterinary anatomical institution as a donation was cut in transverse plane at mid-diaphyseal level. A fitting cylinder introduced into the round medullary cavity provided a rotational hinge and enabled free rotation of both segments about the tibial diaphyseal longitudinal axis (Figure 7A). In the tibia distally, a single plastic pin was glued in transverse axis tangentially along the distal cranial antecochlear tibial axis at the tibial cochlea. The caudal prominences of both tibial condyles (retrocondylar axes) recumbent on the ground of a flat horizontal surface and the diaphysis elevated with the longitudinal axis horizontally allowed the measurement of the tibial torsion angles of the torsion deformity model using a goniometer (universal manual transparent plastic full circle 360° with 1° readout increment, Rulongmeter style; Figure 7B). To evaluate the feasibility and precision of the reference standard, 10 randomly set tibial torsion angles were measured independently by two observers with a goniometer and interobserver agreement was analyzed using the coefficient of variation for repeated measurements. CT scans of the tibial torsion models were performed in a total of 12 different, 6 internal (−) and 6 external (+), hinge rotation setups, manually set at +/−10°, +/−20°, +/−30, +/−40°, +/−50°, and +/−90° deviation from the normal anatomical situation, simulating tibial torsion over a total range of 180°. The DICOM-data were anonymized regarding the torsion angles. Two independent observers, blinded to the preset angles, set the anatomical reference points into the CT images of the various rotated tibial torsion model. When setting the reference points, the operator could not see the resulting angles and measurement results. The developed software templates measured the torsion angles that were compared to the preset angles using Bland–Altman plots and Passing-Bablock regression analysis.

#### 2.3.2. Independency of limb positioning

Independency of tibial positioning on the CT scanner table was evaluated in 20 normal canine left and right tibiae without any signs of osseous deformation or osteoarthritis that were temporarily borrowed from the collection of teaching material of the veterinary anatomical institution. Each tibia was scanned in three different positions. For the first CT scan, all 20 bones were positioned with their longitudinal axis parallel to the z-axis of the scanner. For the second CT scan the tibiae were positioned double oblique to the z-axis having 15° deviation in direction of the x- and y-axes. For the third CT scan the bones were positioned double oblique to the z-axis having 45° deviation to the x- and y-axes. Two independent observers (AB, BS) measured torsion and varus (or valgus) angles in all 60 tibial scans with the developed measurement templates of the software. One operator (BS) repeated all measurements after 6 weeks. To determine precision, inter- and intraobserver agreement was evaluated using Bland–Altman plots. Angular measurements of the obliquely positioned and normal parallel positioned bone scans were compared using subtraction of the results to determine accuracy of the oblique positioned bone measurements using the normal neutral positioned bones as the reference standard.

#### 2.3.3. CT scanning parameter

For all CT scans of the bones, the torsional deformity model and the hind limbs of the clinical canine patients, a helical multi-slice CT scanner with a fixed detector array design (Somatom Definition AS VA48A_02_P12, 64 Excel Ed. software Somaris/7 syngo CT VA48A Siemens Healthcare GmbH, Erlangen, Germany) in helical image acquisition mode was used. Scanning slice thickness was 0.6 mm, tube voltage 120 kV, tube rotation time 0.5–1 s, pitch 0.8 and tube current was variably adjusted according to the size of the object or patient. The reconstructed slice thickness and increment were 0.6 mm. Images were reconstructed using a bone algorithm (deconvolution filter: kernel 60 or 70). The DICOM images of the CT scanner were exported to a network attached storage for further use by medical imaging software.

#### 2.3.4. Precision in clinical CT data

Intraobserver variability (repeatability) and interobserver variability (reproducibility) of the measurements were tested using routinely acquired CT scans of 34 client owned canine patients. These dogs had a clinical diagnosis of patellar luxation and underwent preoperative CT examinations of both hind limbs for routine preoperative assessment and planning of surgical correction using 3D-volume rendering views in addition to the standard radiographic examination between 2012 and 2015. All patients were scanned in a clinical setting preoperatively in general anesthesia that was unrelated to this investigation. The dogs were scanned in dorsal recumbency with extended hindlimbs using foam pads, Velcro strips and tapes. The position was similar to a ventrodorsal pelvic radiograph for canine hip dysplasia screening, having extended coxo-femoral, stifle and tarsal joints with the hindlimbs longitudinal axes parallel to the lumbar spine. In the CT data, two observers (AB, BS) measured all alignment angles of 68 hind limbs of 34 dogs using the developed VoXim® software templates, as outlined above. One operator (BS) repeated all measurements after 6 weeks. Coefficients of variation for repeated measurements were calculated to evaluate the intra- and interobserver agreement.

#### 2.3.5. Statistical analysis

If the angle measurements are independent of the bone positioning in the scanner gantry, then angle calculations based on the 3D coordinates should always provide the same measurement results. Therefore, we subtracted the mean angle measurement results for each angle and bone position in each possible combination (angle results at 0° minus angle results at 15°, angle results at 0° minus angle results at 45°, and angle results at 15° minus angle results at 45°). When subtracting the mean measurement results for each angle between CT datasets with differently positioned bones, the differences in the subtractions should tend to be zero. For calculating the BIAS and its value of p for each angle, the mean differences between the parallel (0°) and each double oblique off-z-axis (15° and 45° off-z-axis deviation) positioning of the tibia on the CT-table in the gantry during scanning were calculated according to Bland and Altman [Bland JM, Altman DG. Statistical methods for assessing agreement between two methods of clinical measurement. Lancet. 1986 Feb 8;1(8476):307–10. PMID: 2868172] (45). Torsion angles measured with the 3D imaging software VoXim® in the CT data were compared with the preset angles in the torsional deformity model using Passing-Bablock regression analysis (46–48) and Bland–Altman plots [Bland JM, Altman DG. Statistical methods for assessing agreement between two methods of clinical measurement. Lancet. 1986 Feb 8;1(8476):307–10. PMID: 2868172] (45). To estimate the repeatability (intra- and inter-observer variability) of tibial angular measurements, the *Coefficient of Variation (CV) for repeated measurements* was calculated according to Bland 2000 [Bland M. An introduction to medical statistics. Oxford University Press 3. Ed. P 269–272] (49). To estimate the intra-observer variability, the *CV for repeated measurements* of the same observer was calculated. To estimate the inter-observer variability, the *CV for repeated measurements* was calculated for the same measurements of different observers. *CVs for repeated measurements* were considered excellent <3%, good <10%, moderate/fair <15%, and poor >15%. The statistical analysis was performed by using the software IBM SPSS 23 and MedCalc 20.111.

## 3. Results

### 3.1. Feasibility

Anatomical reference points at bone surfaces could be set and adjusted using MPR or VR. Anatomical reference points within bones were set using a fixed orthogonal MPR tool. Reference points could be set in any position and obliquity of the bone scans. Reference points set and axes located within bones were projected onto the bone surfaces of VR images and could be viewed from any perspective that enabled 3D overview and enhanced anatomical understanding (Supplementary Figures S1–S6). Reference points set in MPR mode within a single section were projected into the other slices and other planes, and the difference between location (red cross) and projection (yellow cross) was marked with different colors (Supplementary Figure S6B), that also helped to understand the precise anatomical location of the reference point and enabled plausibility tests in different MPR images (Supplementary Figures S1–S6) and from different VR perspectives (Figure 4). When searching for the reference points using VR and MPR, parallel and automatic projection of the reference points within all modes, planes and views facilitated and accelerated the process and reinforced the confidence to set points anatomically correct and precisely, especially in the beginning. MPR mode was also helpful to set reference points within the narrow joint spaces of the tibiaotalar articulation. The application of the technique and use of the templates and tools required initial familiarization and training, as well as continuing practice and the measurements were time consuming, especially in the beginning.

### 3.2. Accuracy in a tibial torsional deformity model

**Figure 8:**
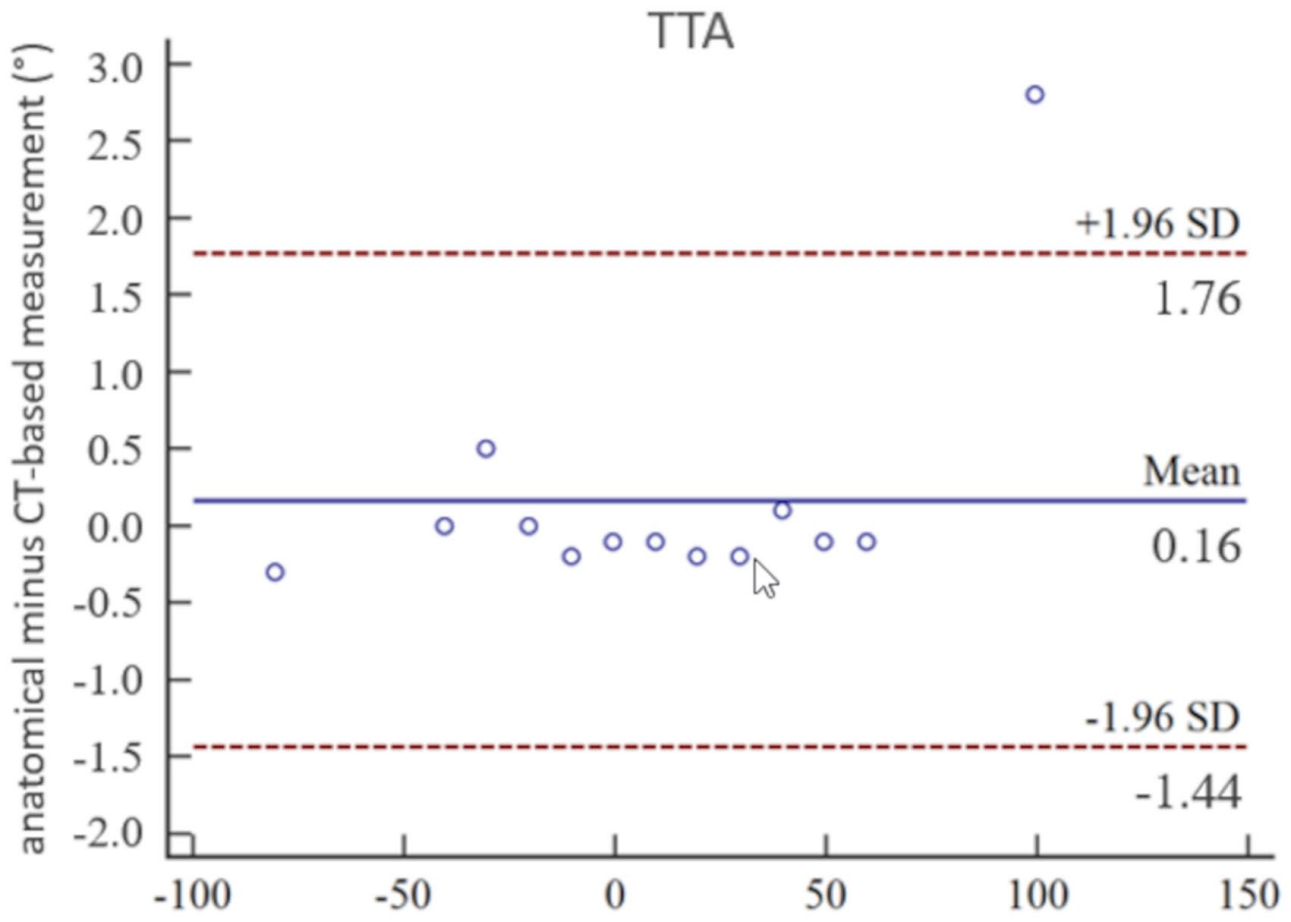
Statistical analysis according to Bland–Altman: accuracy of tibial torsion angle (TTA) measurements expressed by Bland–Altman-Plots of the comparison between anatomical goniometer and CT-based software measurements in a canine tibial diaphyseal torsional deformity model in preset tibial torsion angles. Low mean and standard deviation (SD) demonstrate high correlation between both methods and proves accuracy for the tibial torsion angle measurements.

**Figure 9:**
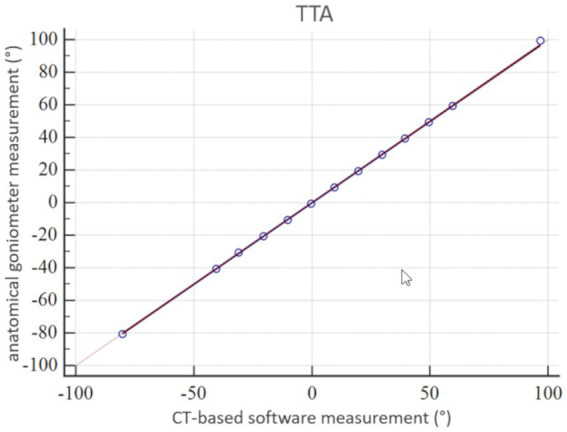
Statistical analysis according to Passing-Bablok: accuracy of tibial torsion angle (TTA) measurements expressed by a scatter plot and regression line of the Passing-Bablok analysis of the comparison between anatomical goniometer and CT-based software measurements in a canine tibial diaphyseal torsional deformity model in preset tibial torsion angles. The 45° straight linear slope of the regression line demonstrates good correlation between both methods and proves accuracy for the tibial torsion angle measurements.

The coefficient of variation for repeated measurements between two anatomical goniometer measurements by two independent observers in the tibial torsion model was 1.77% for the tibial torsion angle (Supplementary Table S1). Therefore, goniometer measurements (Figure 7) were considered a sufficient reference standard for comparison with CT based measurements. In the CT scans of the tibial torsional deformity model in various preset torsion angles the anatomical CT based reference points as described above could be set, the software could calculate the angles and therefore the method was considered feasible. Bland–Altman-Plots of the comparison between anatomical goniometer and CT based software tibial torsion angle measurements revealed a difference of 0.2° as shown in Figure 8. Scatter plots and regression lines of the Passing-Bablok analysis of the comparison between goniometer and CT based software angle measurements are shown in Figure 9. The 45° straight linear slope of the Passing-Bablock regression analysis demonstrates correlation between both methods. Compared with the anatomical goniometer measurements as a reference standard, the CT based software measurements of the tibial torsion angles were considered accurate. The individual results of anatomical goniometer and CT based software tibial torsion angle measurements in the canine tibial diaphyseal torsional deformity model are shown in Table 2.

**Table 2:** Results of accuracy testing of tibial torsion angle measurements in the torsional deformity model of the canine tibia.

Comparison between anatomical (goniometer) and CT-based (software) tibial torsion angle measurements in a canine tibial torsional deformity model in 13 preset diaphyseal torsion angles		
Preset diaphyseal torsion angles (*n* = 13) Antetorsion positive (+) and retrotorsion negative (−)	Tibial torsion angle measurements	
	Bone: macroscopically manually goniometer measured tibial torsion angle	CT-Scan: digitally (software) based angular measurement of the tibial torsion angle
0° (normal)	189.5	189.6
+10°	199.5	199.7
+20°	209.5	209.7
+30°	219.5	219.4
+40°	229.5	229.6
+50°	239.5	239.6
+90°	279.5	276.7
−10°	179.5	179.6
−20°	169.5	169.7
−30°	159.5	159.5
−40°	149.5	149
−50°	139.5	139.5
−90°	99.5	99.8

### 3.3. Independency of limb positioning

In the CT scans with parallel and double obliquely positioned tibiae on the scanner table the anatomical CT based reference points could be found and set, the software could calculate the angles as described above and therefore the method proved feasible in oblique positioning. The results of the test for independency from tibial positioning on the table by double oblique deviation from the z-axis in the scanner gantry expressed by the mean differences between the measurements of various double oblique off-angle scans of the tibia resulted in mean differences less than 1.3° and the individual measurement results are shown in Table 3. Repeated measurements of the double obliquely positioned tibiae by the same person, observer 1 (O1) at two different occasions (observer 1 at occasion 1, O1 (1) and observer 1 at occasion 2, O1 (2), and by two different observers (O1, O2) resulted in coefficients of variation for repeated measurements of 0.24% (intraobserver agreement) and 0.41% (interobserver agreement) for the tibial torsion angle, and 0.3% (intraobserver agreement) and 3.02% (interobserver agreement) for the tibial varus (or valgus) angle (Table 4). The results of all individual tibial measurements are shown in Table 5.

**Table 3:** Results of the tests of independency of tibial positioning: angular measurement accuracy of oblique tibial positioning compared to straight parallel positioning in the CT-scanner gantry.

Mean differences between angular measurement results of various parallel (0°) and varying off-z-axis (15 and 45° deviated) double oblique positioning of the tibiae on the CT-table in the gantry during scanning to test for independency of tibial positioning.			
Tibiae: *n* = 20 Positioning: three times CT-scans: 60	Subtraction of the results of different positioning angles	Mean	*p*-value
Tibial torsion angle (TTA)	TTA (0°) − TTA (15°)	0.188	0.123
	TTA (0°) − TTA (45°)	0.203	0.232
	TTA (15°) − TTA (45°)	0.015	0.919
Tibial varus angle (TVA)	TVA (0°) − TVA (15°)	1.242	0.191
	TVA (0°) − TVA (45°)	0.487	0.093
	TVA (15°) − TVA (45°)	−0.755	0.408

**Table 4:** Results of the tests of independency of tibial positioning: angular measurement precision of oblique tibial positioning compared to straight parallel positioning in the CT-scanner gantry.

Reproducibility of tibial angular measurement results of various parallel (0°) and varying off-z-axis (15 and 45° deviated) double oblique positioning of the tibiae on the CT-table in the gantry during scanning to test for independency of tibial positioning.						
Tibiae: *n* = 20 Positioning: three times CT-scans: 60	Intraobserver Agreement			Interobserver Agreement		
Angle	Coefficient of variation for repeated measurements (%)	Overall mean	95% Confidence interval	Coefficient of variation for repeated measurements (%)	Overall mean	95% Confidence interval
Tibial torsion angle (TTA)	0.24	175.81	0.81 to 3.23	0.41	175.85	0.31 to 0.50
Tibial varus angle (TVA)	0.30	184.17	0.22 to 0.37	3.02	183.94	0.00 to 5.00

**Table 5:** Individual results of observer one for the first time O1(1) and Observer one for the second time O1(2) angle measurement at independend first (1) and second (2) occasion, and of observer 2 (O2), for the accuracy and precision tests of independency of tibial positioning in the CT-scanner gantry.

Results of the test for independency from positioning (*n* = 60) of the tibia (*n* = 20) on the table by double oblique deviation from the z-axis in the scanner gantry: individual angular measurement results of observer 1 (two repeated measurements) and observer 2							
Tibia (*n* = 20) and scan (*n* = 60)	Positioning of the tibia: deviation from z-axis (degree)	TTA			TVA		
		O1 (1)	O1 (2)	O2	O1 (1)	O1 (2)	O2
1.1	0°	171.2	170.9	171.8	185	184.2	185.6
1.2	15°	170.7	170.6	170.1	185.5	184.9	184.6
1.3	45°	170.9	171.1	170.4	185.3	185.2	185.5
2.1	0°	180.3	180.5	180.4	187.9	188	186.9
2.2	15°	179.8	180	180	188.2	188.1	187.3
2.3	45°	180.1	179.7	180.2	187.5	187.8	187.9
3.1	0°	174.9	175.1	174.6	183.7	183.5	183.8
3.2	15°	174.5	174.8	174.1	183.9	183.1	183.8
3.3	45°	175.1	175	174.6	183.3	183.3	183.2
4.1	0°	172.9	171.9	173.5	184.1	184	182.5
4.2	15°	170.1	171.3	172.5	183.7	183.7	181.9
4.3	45°	170.6	171.5	171.5	181.2	182.4	184.3
5.1	0°	175.5	174.3	175.6	188.7	188.6	187.5
5.2	15°	173.9	174.1	175.5	185.6	187.5	188.5
5.3	45°	173.8	174.1	176.1	190.3	189	188.1
6.1	0°	178.8	178.5	179	184.4	184.4	196.4
6.2	15°	178.6	178.7	178.7	183.8	183.7	189.9
6.3	45°	176.1	177.9	176.9	186.9	185.3	191.9
7.1	0°	183.7	183.6	181.3	184.9	185	186.9
7.2	15°	183.1	183.4	181.7	186.1	185.7	186
7.3	45°	181.7	182.9	181	184.1	185.1	184.3
8.1	0°	177.5	177.3	176.9	184.4	184.3	186.5
8.2	15°	177	177.1	177.5	181	182.4	184.9
8.3	45°	177.2	177.2	176.7	182	182.4	183.3
9.1	0°	175.9	175.7	175	184.5	184.4	183.3
9.2	15°	175.3	175.7	176	184.2	184.4	182.5
9.3	45°	175.7	175.4	175.8	185.6	184.9	183
10.1	0°	172.4	172.1	172	184.1	183.9	183.7
10.2	15°	171.2	172.4	171.5	180.3	182.3	183.2
10.3	45°	172.9	172.5	172.3	181	182	183.5
11.1	0°	173.6	173.7	173.5	183.1	183.2	182.8
11.2	15°	174.4	173.8	172.8	181.5	181.7	183.6
11.3	45°	174.9	173.9	174.6	184.3	183.9	182.6
12.1	0°	178.7	178.8	181.4	184.5	184.5	181.8
12.2	15°	178.7	179	180.5	185.9	184.9	182.9
12.3	45°	178.4	178.7	180.1	183.6	183.7	182.3
13.1	0°	177.2	177.1	176.5	187	187.1	186.9
13.2	15°	177.1	176.9	176.4	188.5	188.3	183.8
13.3	45°	177.1	177	176.7	186.9	187	184.7
14.1	0°	176.5	176.5	176.1	180.7	180.9	183.1
14.2	15°	177.5	177	176.5	184.3	182.1	184
14.3	45°	176.7	177	176.5	183.5	182.9	184.1
15.1	0°	172.5	171.9	171.7	179.7	180.1	184.1
15.2	15°	171	171.5	172	181	180.8	185.1
15.3	45°	171.1	171.6	171	180.7	180.7	185.2
16.1	0°	166.3	167	168.9	187.4	187.5	182.7
16.2	15°	166.2	167.1	168.1	186	186.3	184.3
16.3	45°	168.8	167.6	167.9	187.1	186.9	183
17.1	0°	173.9	174.1	174	182.9	182.8	182.6
17.2	15°	174.3	174	174.2	182.5	182.5	183
17.3	45°	174.8	174.3	174.7	182.1	182.3	183.1
18.1	0°	184.3	184.1	184.4	182.1	182.3	182.7
18.2	15°	184.3	184.1	184.7	180.5	180.4	132.2
18.3	45°	185.7	184.6	184.4	180	180.9	183.2
19.1	0°	174.7	175	174.7	184.4	185.1	184.9
19.2	15°	176.6	175.2	175.7	186.3	185	183.2
19.3	45°	175.2	175.1	174.7	180.8	183	183
20.1	0°	179.3	179.3	179.2	185.7	185.7	182.8
20.2	15°	179.3	179	178.9	182.8	184.2	183.4
20.3	45°	178.1	178.6	179.1	181.3	182	182.6

### 3.4. Precision in clinical CT data

In the CT scans of the 34 dogs with patellar luxation the anatomical reference points could be set and the software could calculate the angles. Therefore, we considered the method feasible in clinical patients. Repeated measurements by the same observer 1 (O1) at two different occasions, O1 (1) and O1 (2), and by two different observers (O1, O2) resulted in coefficients of variation for repeated measurements of 2.35% (intraobserver agreement) and 0.60% (interobserver agreement) for the tibial torsion angle, and 2.70% (intraobserver agreement) and 0.97% (interobserver agreement) for the tibial varus (or valgus) angle. All results of the intra- and interobserver agreement calculations are shown in Table 6 and the individual measurement results of each dog and both observers (O1, O2), observer one at two occasions, O1 (1) and O1 (2), are shown in Supplementary Table S2.

**Table 6:** Results of the precision testing based on intraobserver variability (repeatability) and interobserver variability (reproducibility) in a clinical setting in canine patients.

Intra- and interobserver agreement of CT-based tibial angular measurements in a clinical setting in 34 dogs with a clinical diagnosis of patellar luxation that underwent preoperative CT examinations of both hind limbs for routine preoperative assessment and planning of surgical correction						
	Intraobserver Agreement			Interobserver Agreement		
Angle	Coefficient of variation for repeated measurements (%)	Overall mean	95% Confidence interval	Coefficient of variation for repeated measurements (%)	Overall mean	95% Confidence interval
Tibial torsion angle (TTA)	2.35	179.88	0.81 to 3.23	0.60	181.08	0.46 to 0.72
Tibial varus angle (TVA)	2.70	188.57	0.00 to 4.64	0.97	189.18	0.63 to 1.21

## 4. Discussion

### 4.1. Implementation of three-dimensionality

Two-dimensional techniques (2, 12) heavily rely on standardization of positioning and X-ray-beam alignment, or computed tomographic VR-viewing perspective (20). In a 3D VR CT scan, the bone can be virtually rotated. For angular measurements, the bone can be repositioned by the operator. The final selected view and thus the image itself corresponds to the projection plane for the angle measurement. This is difficult to standardize in severely deformed bones (39). We have replaced the visually guided bone positioning with a mathematical definition of bone positioning. We introduced a 3D coordinate system that originated within the tibia. The 3D planes of this bone-centered coordinate system were aligned with the orientation of the anatomical planes of the bone (13, 14). This enabled graphical overlays to be superimposed directly over the tibia. The operator could see anatomical reference points, axes, coordinate system and angles within MPR and VR CT images. The software used the 3D coordinates of the reference points to calculate the angles within geometrically predefined projection planes. Alternatively, an extrinsic coordinate system, for example based on the CT scanner, should also work and lead to the same results.

Minor methodical differences might affect the reference point setting and angular measurement results. Therefore, reference values in dogs might depend on the technique used, as described for the reference values for femoral torsion in human medicine (50, 51). To make the measurements as reproducible as possible, we described the reference points in three orthogonal planes and VR-views. To determine tibial torsion, summation of a proximal and distal tibial CT image that includes the relevant reference points and axes, was described earlier for the canine patient (2, 17, 18, 31, 32, 37). These techniques require two reference points for one axis or tangent, proximal or distal, being located within one transverse single CT slice, which is often not the case in a clinical setting, especially if there is osseous deformity (2, 17, 18, 31, 32, 37). Additionally, mildly oblique positioning causes variation in the appearance of cross-sectional CT images and might be an additional source of error. Therefore, we established a three-dimensional approach that is independent from positioning and does not require to locate two reference points for one axis within one single image. Instead we focused on a technique to set each reference point individually.

Compared to earlier studies (2, 17, 18, 31, 32), we introduced true three-dimensionality by defining the projection plane geometrically, corrected for osseous deformity and oblique positioning. We demonstrated accuracy in a torsional deformity model and precision in the clinical patient. We enhanced the anatomical overview of the cross-sectional CT images by adding a combination of VR and MPR during the process of setting the reference points within the CT data (30). A more recent study used a combination MPR, VR, and fiducial markers to set reference points and standardize viewing perspective for angular measurements of tibial torsion (20). Unfortunately, our software was limited to an orthogonally fixed MPR tool and we assume that a totally free double oblique MPR-tool might help to improve precision or at least simplifies and accelerates reference point setting, especially in cases of osseous deformity and oblique positioning, by correcting and adjusting the image planes to the standardized angles and views. VR visualizes bone surfaces and improves the three-dimensional topographic understanding of the anatomic location of the reference points (15, 36–39). In our study, MPR provided cross-sectional intraosseous information and views that were necessary to set reference points that were located within the bone and MPR was also helpful to set surface points that are intraarticular within narrow joint spaces. Searching for the reference points using VR and MPR parallel, facilitated and accelerated the process and reinforced the confidence to set points anatomically correct and precisely. Superimposition and projections of reference points, axes and angles on MPR- and VR-images enabled a plausibility test in our setting that was helpful, especially in the beginning, because the application of the method, software and use of the templates required initial familiarization, training and continuing practice and is also time consuming, which is a major disadvantage.

### 4.2. Software

DICOM is the technical standard commonly used in veterinary diagnostic imaging (52, 53). Medical device approval and DICOM conformance of the software is an advantage of this study. Since in many countries no medical device approval is currently required in veterinary medicine, other technical platforms could be used for animals, such as CAD (Computer-aided design) software (54, 55). Until an open source software for this method is freely available, we consider cost, dependency on a company and therefore restricted access to the commercial software a limitation of this project. For the future, the development of more intuitive user-friendly software might help to facilitate the setting of the reference points and to improve the precision, especially use and combination of multiple semiautomatic features, like adjustable circle-crosshair-center tools, semiautomatic surface fitting tangents, axis along multiple sections centers or automatically generated ellipses and diameter, as used in other studies in humans and dogs (30, 54, 55). We hope for and encourage veterinary medical imaging software engineers and companies to develop intuitive and user-friendly measurement tools within viewing software that contains measurement capabilities that are truly three-dimensional. Fully automated recognition of reference points and angular measurements are described for the normal canine femur (54, 55). Automatic techniques might also be applicable to deformed bones in the future. Fully automated approaches to detect all reference points independently and to measure all angles automatically by recognition of osseous shapes and surfaces by software, machine learning or deep learning without assistance of a human operator might work in the future. However, the application and feasibility must be demonstrated in canine patients with deformed bones scanned in a clinical setting, not just normal, isolated bones. Manual setting of individual reference points, while time consuming, should allow the operator to set, correct, and adjust reference points even when the shape of a bone is abnormal. We have successfully used the technique on clinical patients. Still, further studies in patients with severe complex deformity, malformation, fracture callus, periosteal reaction, bony remodeling, or periarticular bone formation need to demonstrate the true value of this technique.

### 4.3. Accuracy and precision

We demonstrated the accuracy in isolated tibial torsion in a tibial torsional deformity model with a goniometer as the reference standard. Goniometer measurements with 1° readout increment might not be considered a very precise gold standard, but should be sufficient as a reference standard for torsion angle measurement. Lack of a reference standard to determine accuracy of varus angle measurements is a limitation of the study. A truly three-dimensional technique should be independent from bone positioning. If the angle measurements are independent of the bone positioning in the scanner gantry, then angle calculations based on the 3D coordinates should always provide the same measurement results. Therefore, we compared scans of bones in different oblique positions, to prove three-dimensionality. Based on the small deviations, with mean differences of less than 1.3°, we considered the measurement technique to be accurate and independent of the positioning of the tibia on the CT scanner. Due to the lack of a gold standard for the clinical patients, we demonstrated the precision of the measurement using intraobserver and interobserver variability, sometimes also termed repeatability and reproducibility.

### 4.4. Limitations of the technique

Further studies are necessary to prove that this technique is also accurate in cases with severe bone anomalies that might involve the CT reference points and also in complex three-dimensional osseous deformities with combined variable portions of torsional (rotational), mediolateral (varus or valgus), craniocaudal (ante-/pro- and re-curvature and translational components (8). In this study, we did not measure antecurvature, recurvature, joint orientation angles in the sagittal plane and translational deformity in a three-dimensional approach, but that probably needs to be developed and validated to establish robust three-dimensional angular reference values for canine tibial angles that might vary based on dog type, or might be even breed-specific. A comprehensive evaluation should not only focus on the tibia, but has to involve the canine hind limb as a whole, including all bones and joints and their proportion and contribution to the overall hind limb alignment in a truly three-dimensional way (31, 32, 37).

### 4.5. Anatomical reference points

#### 4.5.1. Tibial torsion angle

Proximally in the tibia, the cross-section of the tibial head is triangularly, cranially the narrow tibial tuberosity and the tibial condyles being wide caudally. A tangent on the caudal canine tibial surface of the tibial condyles proved to be more precise than axes cranially along the tibial condyles, cranially along the tibial tuberosity or the transcondylar axis or between the caudal aspect of the lateral extensor sulcus and the medial collateral ligament protuberance (31, 32). We used the caudal condylar axis proximally and the distal cranial tibial axis for the determination of tibial torsion, because results of a prior study found the measurements of the caudal condylar axis more consistent and reliable than the transcondylar tibial axis (31, 32). Other studies found the transcondylar tibial axis was more reliable than the caudal condylar axis, but the difference was minimal (2, 17). In clinical studies proximal in the tibia the transcondylar and caudal condylar axes were used (18, 37). To determine tibial torsion, distally in the canine tibia the cranial and caudal tibial axis are tangents on the tibial cochlea and are more useful and precise in the canine patient than the bimalleolar axis that is used in human medicine (31, 32). Distally in the tibia, results of earlier studies suggest that use of the distal cranial tibial axis is more reliable than use of the distal caudal tibial axis (2, 17, 31, 32). In these studies, use of the cranial surface tangent resulted in higher precision than use of the caudal surface, likely because the cranial surface of the tibial cochlea is not only more distinct and flatter, but also mediolaterally wider than the caudal surface. Another study found the use of crural torsion using the bimalleolar axis more reliable than tibial torsion (37), but conducted not a direct comparison between both techniques, which was not the subject of our work either. Periarticular new bone formation might change joint margins, where reference points are typically located. The results of studies evaluating angle measurements on normal bones may not be valid in a clinical setting with deformed bones and joints suffering from osteoarthritis. In our equivocal cases, we had the impression that thorough visual inspection of the articular margins especially using sagittal MPR, helped to distinguish between the normal joint margins and periarticular osteophytes. However, future studies are required to assess the influence of severe periarticular osteophytosis or other osseous malformation, deformation, remodeling, exostosis or dysplasia on feasibility, precision and accuracy.

#### 4.5.2. Tibial varus or valgus angle

To determine tibial varus (or valgus) deformity, we set the reference points in the center of the articular surfaces of the lateral and medial tibial condyle proximally and tibial cochlea distally. This is based on proximal and distal tibial joint surface orientation lines on craniocaudal or caudocranial radiographs (1, 3, 5–8, 12, 15, 16) and CT based MPR- (37) and VR-images (38). Based on the slope of the tibial plateau proximally and the depth and concavity of the tibial cochlea distally, tibial positioning, x-ray-beam centering and angulation these proximal tibial reference points are variable on radiographs. Proximally, we choose the centers of the condyles, probably close to the level of the femoral condyle contact points in a normal standing position and therefore at the true level of articulation, but especially paying attention that the tangent was at the same level at the medial and lateral tibial condyle. Different craniocaudal levels medially and laterally change the angle, which is a source of error. Looking at the craniocaudal convexity of the tibial condyles, the lateral tibial condyle is more curved than the medial tibial condyle, that is flatter (19). Therefore, use of the most cranial or caudal point would change the angles too. This process might be improved in the future by a semiautomatic area midpoint calculation or center fitting tool. Distally, we choose the deepest and therefore the most proximal and axial points in the tibial cochlea. Setting a point into the center of an oval curved area by visual inspection is a source of variation. Use of abaxial medial and lateral joint margins would increase the distance between the reference points and therefore increase precision, but these points might not truly represent the level and angle of articulation. Reference points at the abaxial medial and lateral articular margins are easier to set and further away from each other, but are not true articular contact points. Medially and laterally, there might be differences between articular center and margin, based on depth of joint concavity and periarticular elevations. The medial and lateral malleolus might protrude differently based on normal variation and points at joint margins are prone to alteration by periarticular new bone formation due to osteoarthritis. Reference points at articular surface centers are indirectly estimated or calculated midpoints and these could be altered by subchondral bone defects, but these are likely not as common as periarticular new bone formation and also easier to recognize. Instead of the articular centers or the abaxial articular margins medially and laterally, use of the most cranial or caudal articular margins would also be an alternative. The cranial margins of the tibial condyles are difficult to set, because they merge with the tibial tuberosity. The caudal margins are the reference points of the tibial torsion angle.

#### 4.5.3. New anatomical reference points

We used different reference points for the determination of tibial torsional and varus (or valgus) deformities. This seems not reasonable from an anatomical perspective, but is caused by technical reasons. Imaging in the canine patient is defined by standardized positioning and not by standing posture of the animal. Transverse CT images have higher in-plane resolution in x- and y-axis than along the z-axis. Older studies did not use MRP and had probably only transverse slices with anisotropic voxel geometry (2, 17, 31, 32). The error due to partial volume effects along the z-axis at the joint surface level equals the slice thickness, that was not smaller than 1 mm in the past. Therefore, it might be useful, to reevaluate results of older studies with modern CT scanner technique. Today having submillimeter high-resolution CT scanner with high-resolution MPR does not only allow to improve precision, but might also allow the use of other or additional reference points for the future. Instead of the use of cranial and caudal bone surface points, tibial torsion might be calculated using the articular surface center points. Use of standardized joint rotation centers, for example standardized femorotibial contact points of a dog in a standing posture seems an anatomical approach, that might be used reasonable for both, the calculation of tibial varus (or valgus angles) and also for the tibial torsion angle.

#### 4.5.4. Mathematical definition of projection planes

Skew lines require projection into a common shared plane to enable angular measurements. Definition of these planes might alter the results, depending on the type of deformation. To measure torsional deformities, projection planes are oriented transverse, orthogonally to the longitudinal axis of the bone. There are several alternative ways to define transverse projection planes based on various axes, for example the proximal or distal tibial longitudinal axes or their bisecting angle or mid-diaphyseal longitudinal axes based on mid-diaphyseal reference points. These differences might not matter in normal straight bones, but might play a role, if there are additional craniocaudal or mediolateral deformities of the diaphysis (39). Therefore, we choose a total tibial longitudinal axis with a proximal and distal diaphyseal reference point with the idea to make the method robust for diaphyseal deformities, which is not proven yet. To determine varus and valgus deformity we choose a dorsal plane that was defined by the proximal and distal tibial shaft centers (=total tibial longitudinal axis) and parallel translation of the proximal tibial joint orientation line (=proximal tibial dorsal axis, PTDAx) to the total tibial longitudinal axis (TTLAx). Alternatively, the distal tibial joint orientation line (=distal tibial dorsal axis, DTDAx), the proximal caudal tibial retrocondylar axis (TRCAx) or the bisecting angles between the distal and proximal tibial mediolateral joint surface axes could be used. Also, the tibial longitudinal axis could be defined differently and therefore the dorsal plane. In normal bones this might cause only minor differences, but these alternatives might play a role in cases of severe or complex combined deformations along various axes (39).

In conclusion, we developed a method to measure canine tibial torsional and varus or valgus deformities, that calculates in 3D space, and we demonstrated its accuracy in a torsional deformity model, and its precision in CT data of clinical patients.

## Data availability statement

The raw data supporting the conclusions of this article will be made available by the authors, without undue reservation.

## Ethics statement

Ethical review and approval was not required for the animal study because clinical CT data of canine patients in veterinary hospital were scanned unrelated to this research. Written informed consent for participation was not obtained from the owners because clinical CT data of canine patients in veterinary hospital were scanned prior and unrelated to this research. Clinical CT data were retrieved from the image archive. For the experimental scans of the bones, the specimens were temporarily borrowed from the Veterinary Institute. The bones came from donations by animal owners from the cadavers left after the death of their animals.

## Author contributions

Conception and study design, development of methodology, measurements and data acquisition (operator 2, observer 2), analysis and interpretation as well as draft, revisions, approval and submission of the article was the contribution of AB. Measurements and data acquisition (operator 1, observer 1), analysis and interpretation of data as well as final approval of the article was the work of BS. Experimental and clinical CT scans as well as final approval of the completed article were the contributions of MZ. SR selected and calculated the statistical tests, revised the article for intellectual content and approved the final article. Supervision, clinical patient acquisition, orthopedic examinations and surgeries in the dogs with patellar luxation, revision of the article for intellectual content and approval of the final article was the contribution of AM-L.

## Conflict of interest

The authors declare that the research was conducted in the absence of any commercial or financial relationships that could be construed as a potential conflict of interest.

## Publisher’s note

All claims expressed in this article are solely those of the authors and do not necessarily represent those of their affiliated organizations, or those of the publisher, the editors and the reviewers. Any product that may be evaluated in this article, or claim that may be made by its manufacturer, is not guaranteed or endorsed by the publisher.
