# Supplementary figures and images for: Three-dimensional computed tomographic angular measurements of the canine tibia using a bone-centered coordinate system
Source: Front Vet Sci. 2023 May 30;10:1154144. doi: 10.3389/fvets.2023.1154144 (PMC10267710; doi:10.3389/fvets.2023.1154144)

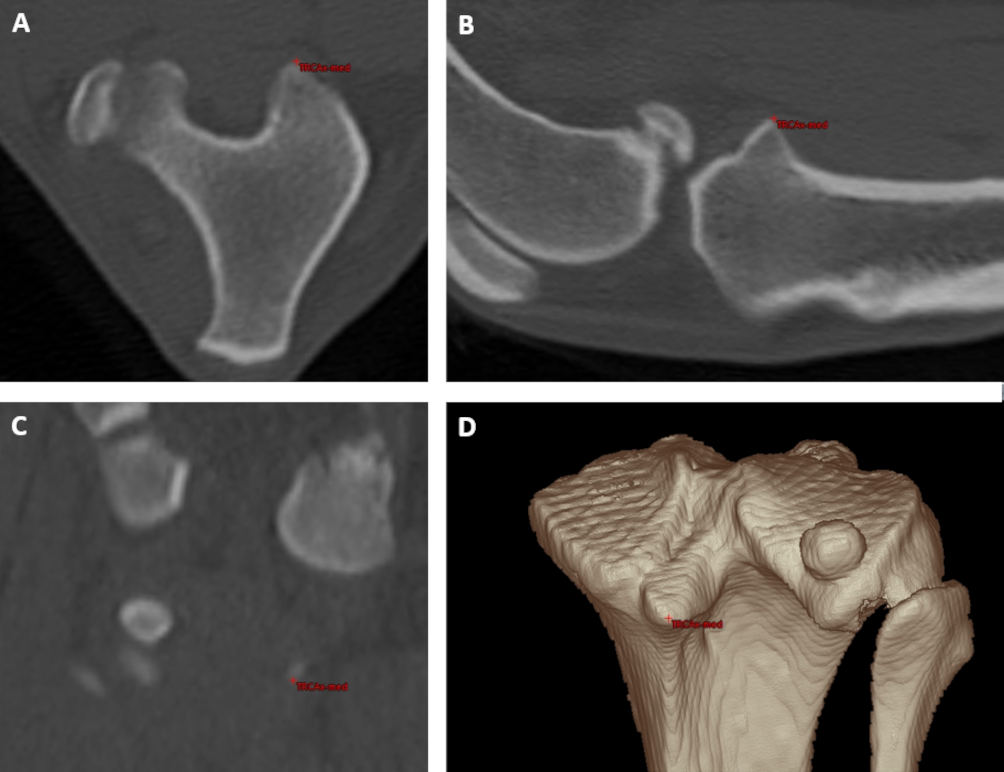

Supplement: Supplementary file 3 [file Image_1.TIFF]

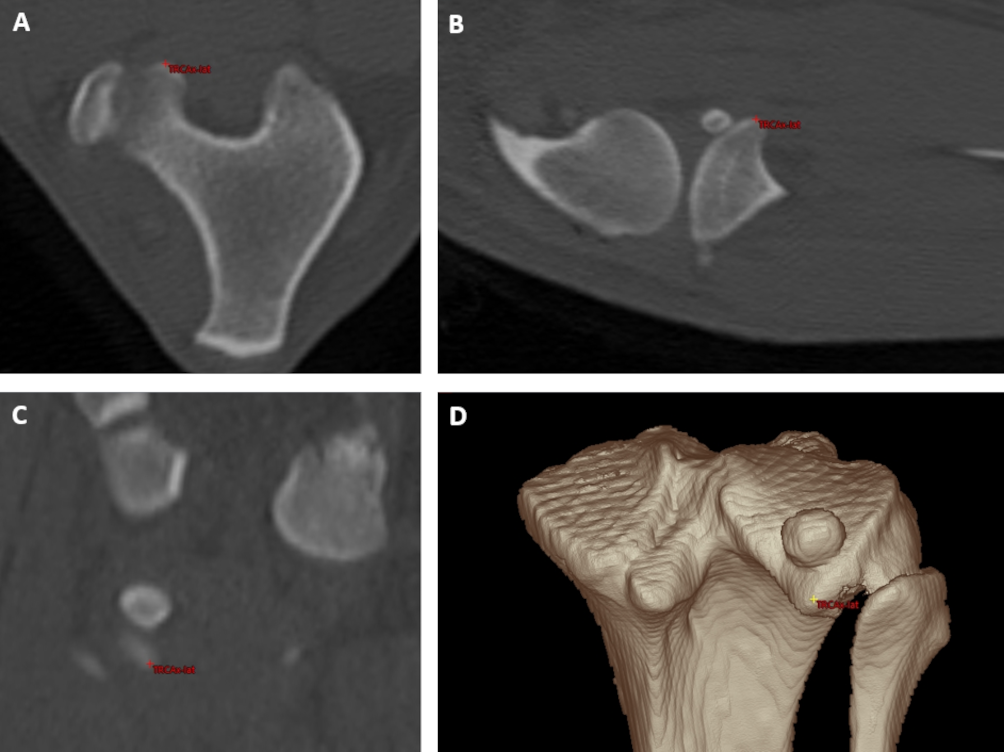

Supplement: Supplementary file 4 [file Image_2.TIFF]

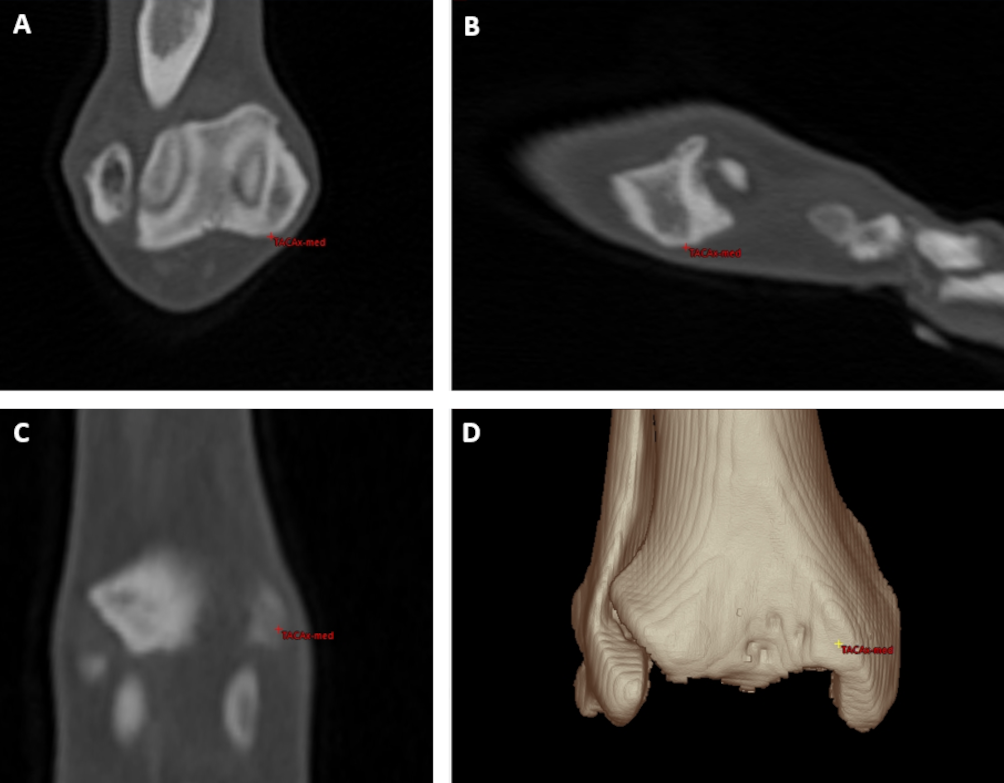

Supplement: Supplementary file 5 [file Image_3.TIFF]

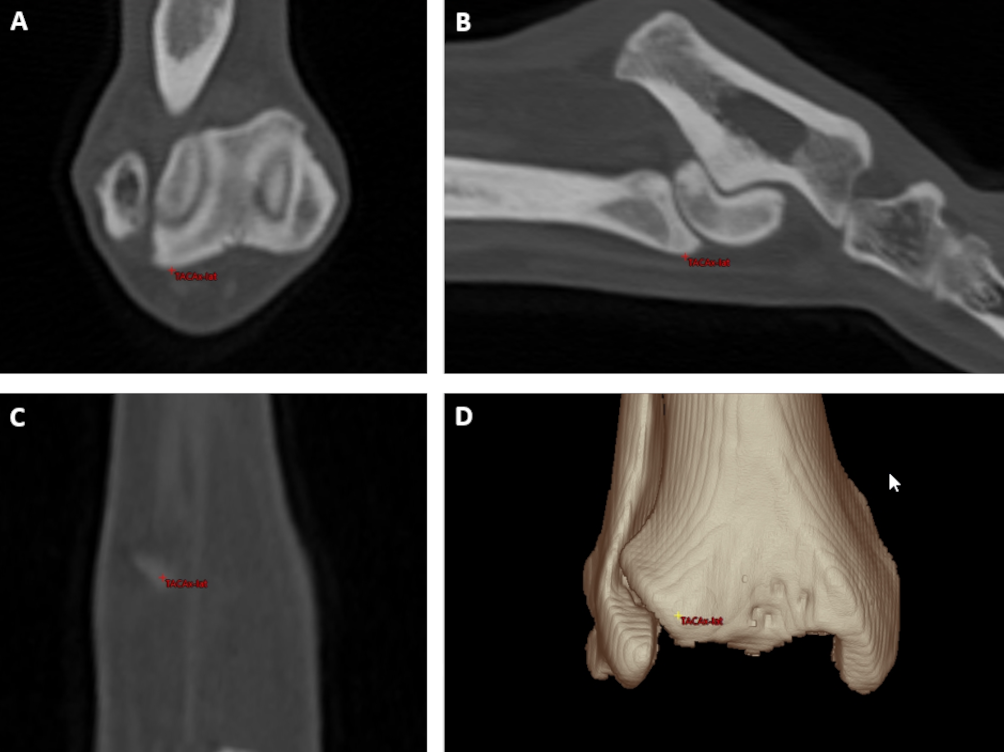

Supplement: Supplementary file 6 [file Image_4.TIFF]

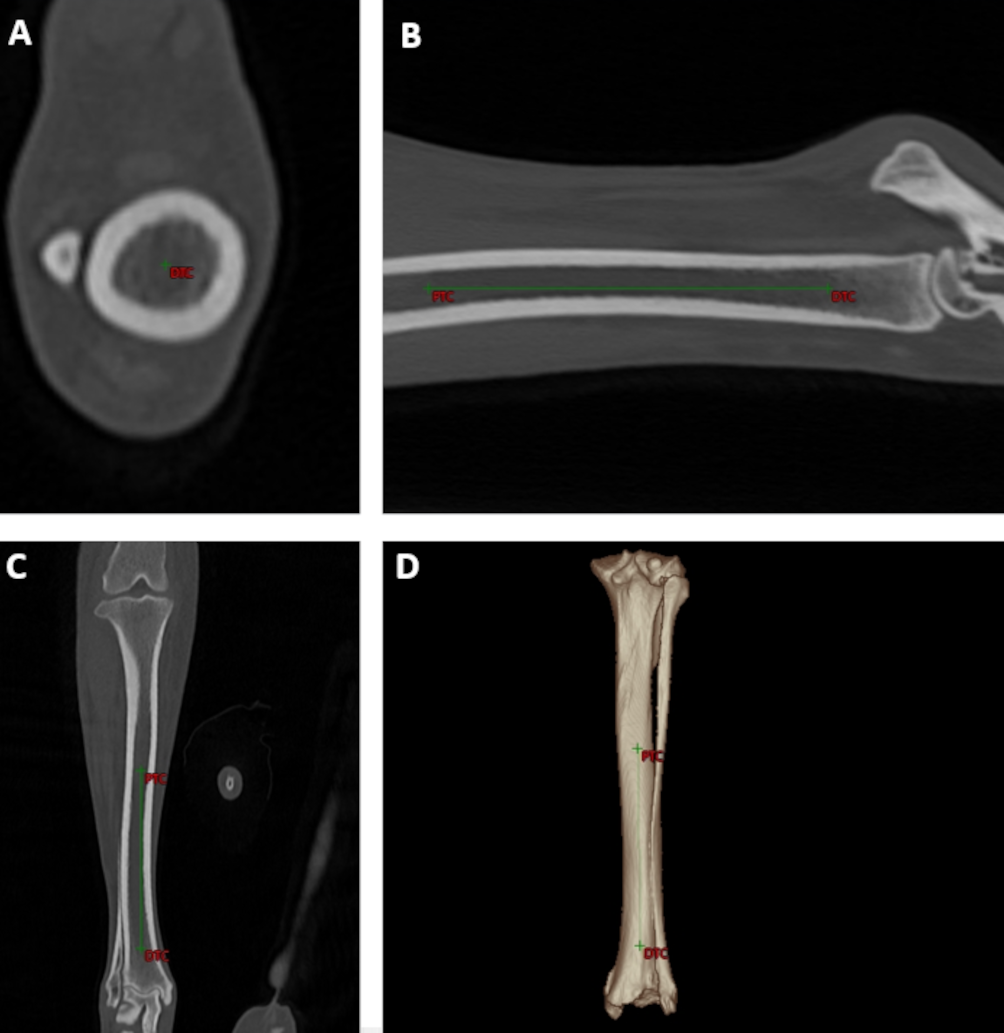

Supplement: Supplementary file 7 [file Image_5.TIFF]

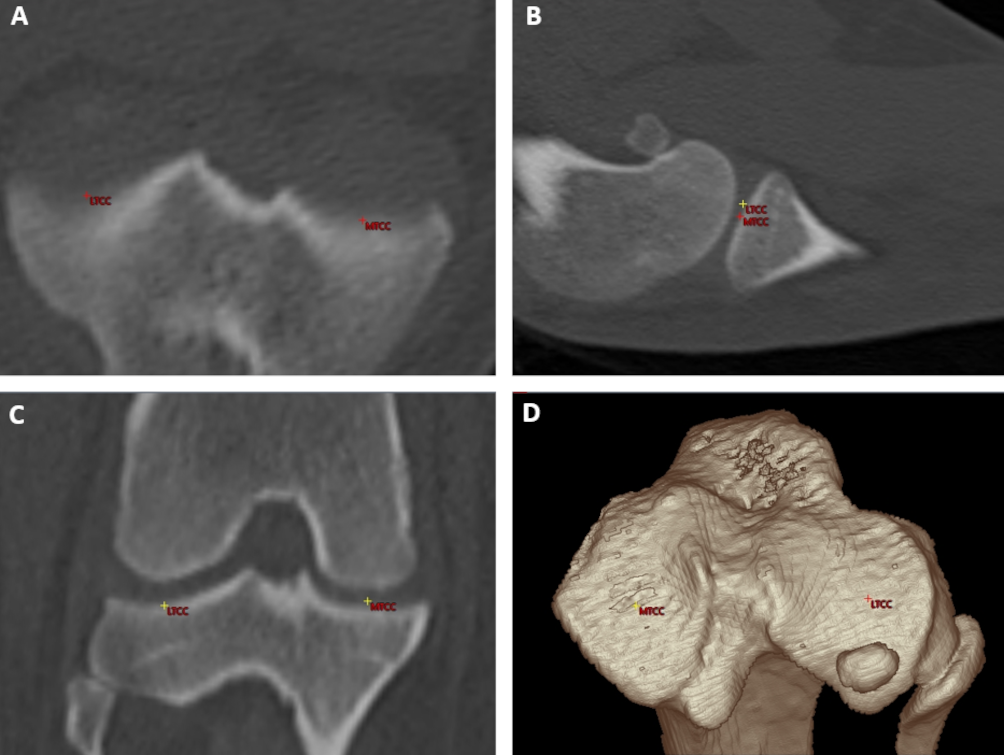

Supplement: Supplementary file 8 [file Image_6.TIFF]

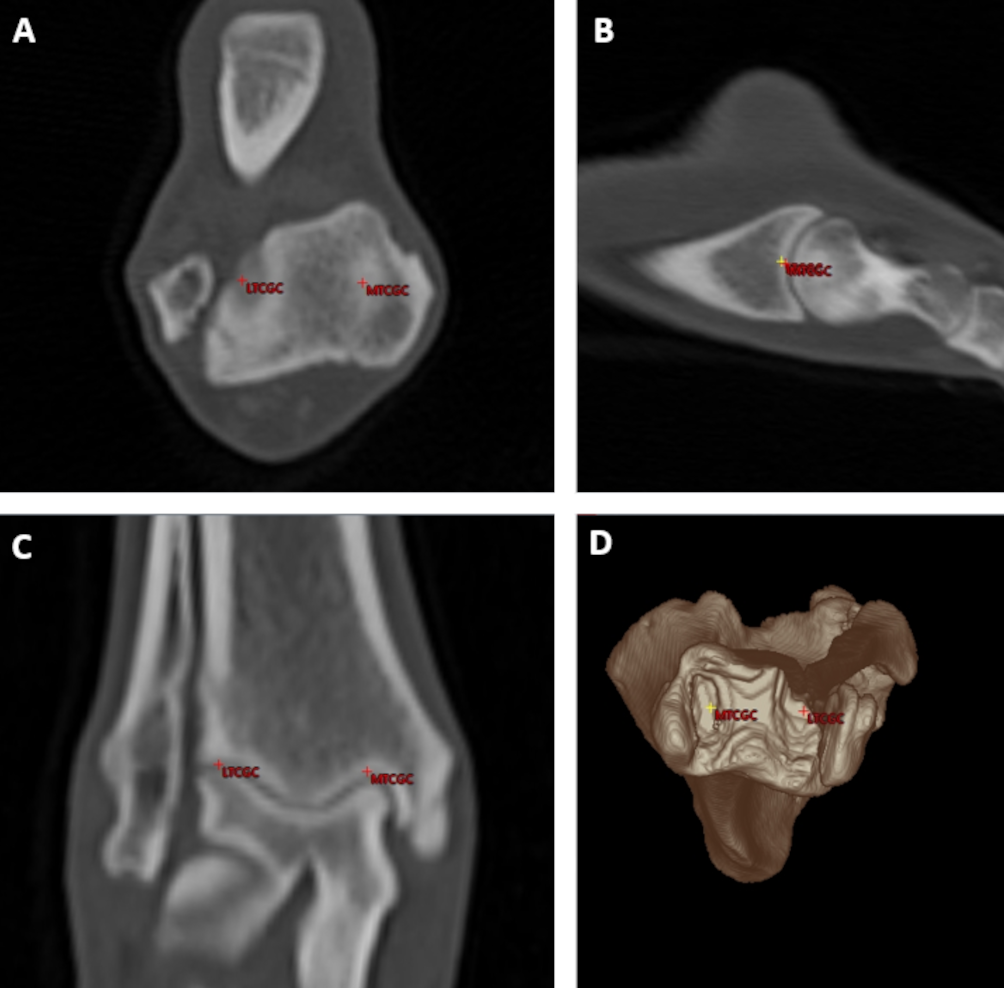

Supplement: Supplementary file 9 [file Image_7.TIFF]
